# Evaluation of Motor Complications in Parkinson's Disease: Understanding the Perception Gap between Patients and Physicians

**DOI:** 10.1155/2021/1599477

**Published:** 2021-12-22

**Authors:** Hiromu Ogura, Ryoko Nakagawa, Miwako Ishido, Yoko Yoshinaga, Jun Watanabe, Kanako Kurihara, Yuka Hayashi, Koichi Nagaki, Takayasu Mishima, Shinsuke Fujioka, Yoshio Tsuboi

**Affiliations:** ^1^Department of Neurology, Faculty of Medicine, Fukuoka University, 7-45-1 Nanakuma, Johnan-ku, Fukuoka-shi, Fukuoka 814-0180, Japan; ^2^Medical, AbbVie GK, 3-1-21 Shibaura, Minato-ku, Tokyo 108-0023, Japan

## Abstract

**Background:**

Patients with Parkinson's disease (PD) receiving levodopa treatment often report motor complications including wearing-off (WO), dyskinesia, and morning akinesia. As motor complications are associated with a decrease in patients' quality of life (QoL), it is important to identify their occurrence and commence immediate management. This study investigated whether differences in the perception of motor complications exist between patients and their physicians in routine clinical practice.

**Methods:**

After an Internet-based screening survey, questionnaires were distributed to physicians and their patients in Japan. The 9-item Wearing-Off Questionnaire (WOQ-9) was used to objectively assess the presence of WO; patients with WOQ-9 scores ≥2 were considered to have WO. McNemar's test was used to compare physician assessment versus WOQ-9 scores, patient self-awareness versus physician assessment, and patient self-awareness versus WOQ-9, separately. Morning akinesia and dyskinesia were assessed by both physician assessment and patient self-awareness with McNemar's test. QoL was assessed using the 8-item Parkinson's Disease Questionnaire (PDQ-8) with the Wilcoxon rank-sum test.

**Results:**

A total of 235 patients with PD and their 92 physicians participated in this survey. A significant discordance was observed between the WOQ-9 and physician assessment of WO (67.2% vs 46.0%; *p* < 0.0001). Furthermore, patient self-awareness of WO was 35.3% (*p* = 0.0004, vs physician). Morning akinesia (patient, 58.7%; physician, 48.9%; *p* = 0.0032), dyskinesia (patient, 34.0%; physician, 23.4%; *p* = 0.0006), and bodily discomfort (patient, 25.0; physician, 0.0; *p* = 0.0102) of QoL were underrecognized by physicians.

**Conclusions:**

This study investigated differences in the perception of WO between patients with PD and their physicians in routine clinical practice and highlighted that patients have a low awareness of the symptoms of WO compared with physician assessments and WOQ-9. Conversely, morning akinesia, dyskinesia, and bodily discomfort were underrecognized by physicians.

## 1. Introduction

Parkinson's disease (PD) is a complex, heterogeneous, neurodegenerative disease [1] characterized by progressive motor symptoms, including tremors, rigidity, and bradykinesia, with postural instability often appearing as the disease progresses [2]. PD is also associated with various nonmotor symptoms, such as cognitive and psychiatric disturbances, autonomic dysfunction, sleep disorders, pain, fatigue, and olfactory dysfunction [1]. As PD is an incurable progressive condition, the aim of treatment is to control symptoms for as long as possible, improve mobility and function, and maintain the overall quality of life (QoL) of patients [3].

International guidelines, including those of the Japanese Society of Neurology, recommend either levodopa or dopamine agonists for the symptomatic treatment of PD [4, 5]. As the disease progresses, most patients receiving long-term treatment with levodopa develop motor complications [6, 7]. Frequently, the first motor complication to appear is the “wearing-off” (WO) phenomenon, where the symptomatic benefit of a certain dose of levodopa is not maintained until the next dose. Other motor complications include dyskinesia in the intermediate stage and complex motor fluctuations in the advanced stage [7]. The occurrence of WO increases gradually, with the majority of patients with PD experiencing WO within 10 years after the initiation of levodopa therapy [8]. WO is also common in the early stages of PD. Results from a survey conducted in an Asian population showed that 29% of patients who received levodopa for <1 year experienced WO, which increased to 68.3% after >10 years of treatment [9]. Because the occurrence of WO is associated with a decrease in patient QoL [8], it is important to identify its occurrence and commence immediate management.

The recognition of WO symptoms, which has typically relied on physician judgment, can be challenging, especially in the early stages of the disease, and may therefore be overlooked in routine clinical practice [8, 10, 11]. Results from a survey that explored issues surrounding WO and QoL found that although the majority of patients (87%) stated that they understood what “WO” meant, only 30% of patients provided correct answers on further questioning [12]. Because the early detection of initial motor complications is essential for timely assessment, to optimize therapy and to improve quality of care and patient outcomes, several questionnaires have been developed to improve the recognition of WO. The 32-item Wearing-Off Questionnaire (WOQ-32) consists of a checklist of symptoms for patients, with questions on whether their symptoms improve with medication [10]. For practical reasons, this questionnaire was adapted to a 19-item questionnaire (WOQ-19) and, later, to a 9-item questionnaire (WOQ-9), which had the same features but was more suitable for routine clinical use [13, 14].

WO is underestimated by physicians. Results from two trials have shown that patients identified the presence of WO on the basis of the self-administered WOQ-9 [11] and WOQ-19 [8] more frequently than did physicians during neurological evaluation. Although several studies have examined the potential differences in the perception of WO between patients and physicians [8, 10, 14], detailed investigations, particularly in Japan, are lacking. We aimed to explore whether there were any differences in the perception of WO, as well as other health-related outcomes, between patients with PD and their physicians, as assessed in routine clinical practice in Japan.

## 2. Patients and Methods

### 2.1. Study Design

This was an observational, cross-sectional study conducted in Japan. It complied with the local laws and regulations and was performed in accordance with the guidelines for Good Pharmacoepidemiology Practices in noninterventional studies and the Japan Ethical Guidelines for Medical and Health Research Involving Human Subjects. This study was approved by the external institutional review board of the nonprofit organization MINS (Tokyo, Japan; approval # 180205).

### 2.2. Study Methods

Physicians registered in the INTAGE physician panel (INTAGE Inc., Tokyo, Japan) were eligible to participate in the study. Initially, a preliminary Internet-based screening survey was conducted to select physicians who had treated more than five patients per month. The study consisted of two sequential periods. First, a screening was conducted to determine physician eligibility and interest in participating in this study over the Internet. Second, the main survey was conducted, which consisted of instructions for physicians (Supplementary Table 1), patient demographics and disease characteristics, WOQ-9 for completion by patients, and the 8-item Parkinson's Disease Questionnaire Summary Index (PDQ-8 SI) for completion by both physicians and patients (Supplementary Tables 2–4). No specific questions on adverse events were included in the survey.

The target number of participant physicians was set as described in “Sample size calculation and statistical analyses,” and physicians having a large number of patients available to answer the questionnaires and experience to participate in the survey were selected on high priority. Selected physicians received the sealed patient questionnaires, which were given to patients who fulfilled the inclusion criteria of the study. Physicians did not know the contents of the patient questionnaire. Each physician was required to recruit at least three patients during the study period. Both patients and their physicians were required to complete the questionnaires within 14 days of the clinic appointment. The completed patient and physician questionnaires were collected separately by mail. The questionnaires returned by physicians and patients were physically collated to ensure appropriate pairing of physicians and patients.

### 2.3. Eligibility

Adult patients with a confirmed diagnosis of idiopathic PD who had received any treatment for PD continuously for >1 year and who were willing and able to provide informed consent were included in the study. Physicians who had treated at least five patients per month and who were willing and able to provide informed consent were also included in the study.

Patients unable to complete the questionnaires by themselves and who had no support for questionnaire completion from caregivers were excluded from the study. Patients and physicians who were unable to submit the questionnaires within 14 days after the clinic appointment were also excluded.

### 2.4. Study Endpoints

The primary endpoint was to compare the proportion of patients identified as having WO on the basis of WOQ-9 and by physician assessment. Patient self-awareness of WO was assessed by the question “Normally, do symptoms emerge before the next medicine (“wearing-off phenomenon”)?” (Supplementary Table 4, Q13). WOQ-9, which was answered by patients, is described in Supplementary Table 4, Q21. Physician assessment of WO was assessed by the question “Has “wearing-off” emerged in the patient as of present?” (Supplementary Table 3, Q4). In our previous study [15], the Japanese version of WOQ-9 with one positive response showed high sensitivity (94.1%), low specificity (39.2%), and reasonable accuracy (70.0%) in diagnosing WO. With more than two positive responses, the sensitivity of WOQ-9 decreased from 94.1% to 87.1%, while the specificity increased from 39.2% to 72%; the accuracy of detecting WO also increased from 70.0% to 81.4%. Therefore, patients with WOQ-9 scores ≥2 were considered to have WO in this study.

Secondary endpoints included a comparison of the differences between patient self-awareness of WO and physician assessment of WO, an assessment of the differences in the reporting of morning akinesia and dyskinesia between patient self-assessment and physician judgment, and an evaluation of the reported differences in QoL between patient self-assessment and physician judgment, as assessed using PDQ-8 SI.

### 2.5. Sample Size Calculation and Statistical Analyses

It was estimated that recruitment of approximately 600 physicians would be feasible for this study. Based on a cooperation rate of 20%, each physician had to recruit at least three patients. The return rate of the matched pairs of questionnaires was estimated to be 60%. Based on these assumptions, the number of patients required for appropriate statistical power was approximately 210: 600 × 20% (cooperation rate) × 60% (paired) × 3 patients (per physician) = 216. A previous study reported that 15.1% of patients were identified as having WO based on WOQ-9 and were not identified by neurologists, whereas 2.0% of patients were identified as having WO by neurologists and were not identified based on WOQ-9 even in specialized centers [11]. Assuming discordance rates of 15% and 2%, a sample size of 76 was considered sufficient to detect differences in the reporting of WO between physicians and patients using McNemar's test with a type I error rate of 5% and a power of 80%. Therefore, a total of 210 patients and 70 physicians were required to be enrolled in the study. Descriptive statistics were used to describe patient demographic data of all patients who met the inclusion criteria and returned their questionnaires.

The primary endpoint was evaluated using McNemar's test. In addition, the kappa coefficient and 95% confidence interval were determined for the proportion of patients identified with WO. Secondary endpoints that included differences in the reporting of morning akinesia and dyskinesia between patient self-awareness and physician assessment were evaluated using McNemar's test. The difference in PDQ-8 SI score, as assessed by patients and physicians, was evaluated using the *t* test or the Wilcoxon rank-sum test after determination of data distribution using the Shapiro–Wilk test.

## 3. Results

### 3.1. Patient Demographics and Baseline Clinical Characteristics

In this study, questionnaires were sent to 96 physicians, of whom 93 (96.9%) responded. Among patients, questionnaires were sent to 279 patients, of whom 237 (84.9%) responded. Overall, 237 patients with PD and 92 physicians were enrolled in the study from multiple centers across Japan between April 2018 and October 2018. Two patients were excluded as they were not taking any medications for PD; therefore, a total of 235 patients and 92 physicians participated in the study.

Table 1 shows the demographics and clinical characteristics of the patient population. The mean age of patients was 74.0 years, and 51.9% were women. The average age at PD diagnosis was 65.3 years, and the mean duration of PD was 8.5 years. The majority of patients were in Hoehn and Yahr (H&Y) stage 3 (25.5%) or stage 4 (26.8%); 9.4% of patients were in H&Y stage 5.  Table 2 shows the physician demographics. Most physicians were aged between ≥50 and <60 years (41.3%), and 94.6% were men. The proportion of physicians belonging to university hospitals, public hospitals, and other hospitals was 6.5%, 14.1%, and 43.5%, respectively, whereas 34.8% of physicians were affiliated to clinics. Several physicians were affiliated to departments of neurology (56.5%) and to the Japanese Society of Neurology (65.2%).

### 3.2. Presence of WO

WOQ-9 (Supplementary Table 4, Q21) identified the signs and symptoms of WO in 67.2% of cases (Table 3). WO was observed in 46.0% of cases by physician assessment and in 35.3% of cases by patient self-awareness (Table 3). Discordance between patient self-awareness (Supplementary Table 4, Q13) and physician assessment (Supplementary Table 3, Q4) was observed in 49 cases (20.9%; *p* = 0.0004); 37 patients (15.7%) underrecognized and 12 patients (5.1%) overestimated their WO compared with the physician assessment (Table 4). Discordance between patient self-awareness and WOQ-9 was observed in 99 cases (42.1%; *p* < 0.0001, sum of 12 and 87 cases). On the other hand, discordance between physician assessment and WOQ-9 was observed in 92 cases (39.1%; *p* < 0.0001, sum of 21 and 71 cases; Table 5). Mean (standard deviation [SD]) duration of WO per day assessed by patients was 2.95 (2.52) hours (Supplementary Table 5).

### 3.3. Presence of Morning Akinesia

Morning akinesia was observed in 115 patients (48.9%) by physician assessment and in 138 patients (58.7%) by patient self-awareness (*p* = 0.0032; Table 3). The presence of morning akinesia was reported by patients but not physicians in 42 cases (17.9%) and by physicians but not patients in only 19 cases (8.1%; Table 4). Mean (SD) duration of morning akinesia per day assessed by patients was 1.32 (1.82) hours (Supplementary Table 5).

### 3.4. Presence of Dyskinesia

Differences in the presence of dyskinesia were observed between physician assessment (*n* = 55, 23.4%) and patient self-awareness (*n* = 80, 34.0%; *p*=0.0006; Table 2). The presence of dyskinesia was underreported by physicians in 39 patients (16.6%); in contrast, only 14 patients (6.0%) were not self-aware of their dyskinesia (Table 4). Mean (SD) duration of dyskinesia per day assessed by patients was 3.47 (4.00) hours (Supplementary Table 5).

### 3.5. QoL Assessed by Patients and Physicians (PDQ-8 SI)

No significant differences were observed between physicians and patients in the assessment of QoL (based on PDQ-8 SI and subdomain scores) with the exception of bodily discomfort, which was poorly recognized by physicians (patient median, 25.0 vs physician median, 0.0; *p* = 0.0102; Table 6). The PDQ-8 SI score as assessed by patients in whom the presence of WO was not determined by patient self-awareness, physician assessment, or WOQ-9 was 25.6; that score as assessed by physicians was 28.5 (Supplementary Table 6). On the other hand, even when patients did not consider themselves as experiencing WO, the population that both physicians and WOQ-9 assessed as having WO showed worse QoL (PDQ-8 SI scores were 37.7 and 39.4, as assessed by patients and physicians, respectively).

The population in which patients themselves were aware of morning akinesia, but physicians did not assess the presence of morning akinesia, showed worse QoL (PDQ-8 SI scores of 39.9 and 43.2, as assessed by patients and physicians, respectively) compared with the population in which both patients and physicians indicated the absence of morning akinesia (23.5 and 20.7, respectively; Supplementary Table 7).

## 4. Discussion

Although levodopa is still recognized as the most effective medication for PD, long-term treatment is often associated with motor complications [16], which impair daily living and have a negative impact on patients' QoL. Since WO is generally the first motor complication to develop, its early identification is of great importance for the timely optimization of therapy [8].

The overall prevalence rate of WO by patient recognition in this study was 35.3% (Table 3), which was nearly identical to that reported in another Japanese survey in 407 patients from the Japan Parkinson Disease Association, where 36% of patients, with a mean age of 69 years and disease duration ranging from 3 to 9 years, reported WO [17]. Results from this study highlighted the low awareness of WO among patients compared with both physician assessment and WOQ-9, which suggests that patients do not easily recognize the early signs of WO. In line with previous reports [10, 11], our study confirmed that differences in the perception of WO exist between patients and their physicians, as evidenced by the fact that WO was observed in 46.0% of patients by physician assessment and in 35.3% of patients by patient self-awareness. On the other hand, WOQ-9 could detect WO with high sensitivity. WOQ-9 identified WO in 67.2% of patients compared with physician assessment, which identified WO in only 46.0% of patients (Table 3). We considered patients with WOQ-9 scores ≥2 as having WO based on our previous study [15], which showed that the sensitivity and specificity for WOQ-9 scores ≥2 were 87.1% and 72.2%, respectively, whereas those for WOQ-9 scores ≥1 were 94.1% and 39.2%, respectively. Therefore, WOQ-9 scores ≥2 might help avoid false-positive findings and maintain high sensitivity. Our previous findings may be attributable to the clear and concise nature of WOQ-9, which makes it quick and simple to use. In contrast, busy physicians may not always have sufficient time to adequately ask their patients about potential symptoms of WO [10]. Therefore, WOQ-9 can be an effective screening tool that aids in the diagnosis of WO in patients with PD. On the other hand, the specificity and sensitivity for WOQ-9 scores ≥2 were 72.2% and 87.1%, respectively [15], which are not sufficiently high to identify WO symptoms. These rates were calculated based on the clinical judgment of WO by physicians with >7 years of experience in treating PD patients. Overall, 158 patients were evaluated as having WO by WOQ-9 (Table 3). Therefore, 131 patients in this survey were judged by experienced neurologists as having WO based on this calculation. Furthermore, the number of patients judged as having WO in this survey by physician assessment (*n* = 108; Table 3) was still lower than the calculated value. This difference may be attributable to the differences in physician experience, and it should be noted that assessment by experienced neurologists remains the gold standard in the diagnosis/identification of WO. In this survey, 65.2% of physicians were members of the Japanese Society of Neurology. Thus, approximately two-thirds of physicians were considered to be neurologists, and the remaining were nonspecialists. One explanation for the lower recognition of WO by physicians could be that a considerable percentage of PD patients were being treated by non-neurologists in Japan. WOQ-9 may be an effective tool for the diagnosis of WO by nonspecialists.

Morning akinesia, which is common in patients with PD, not only causes significant disability but also has a negative impact on patients' QoL [18, 19]. Indeed, results from a European, multicenter, observational study showed that up to 60% of patients experience morning akinesia, which prevents them from performing morning routines [19]. In our study, patients reported morning akinesia to a greater extent than did physicians, with 58.7% of patients reporting morning akinesia compared with 48.9% of physicians (Table 3). This finding is in line with the results of an Italian survey in 151 patients, which showed that 64.2% of patients reported morning akinesia [20]. Taken together, these findings suggest that more patients reported the presence of morning akinesia and are therefore more likely to discuss any concerns with their physicians [18]. Moreover, results from our study showed that patients were far more likely to report morning akinesia than WO. In contrast, physicians reported similar rates of recognizing akinesia and WO (48.9% and 46.0%, respectively). Consequently, there may be a perception among some physicians that patients are able to identify the early symptoms of WO as easily as those of morning akinesia; however, owing to the heterogeneity of the signs and symptoms of WO, patients frequently underrecognize WO, a finding that was observed in our study [10].

Episodes of dyskinesia pose a major challenge in the long-term management of patients with PD [21]. It is therefore unsurprising that troublesome dyskinesia was reported by 34.0% of patients, an observation that corroborates findings from previous studies where treatment-induced dyskinesia occurred in 28.0%–40.0% of patients [6, 21]. Both dyskinesia and morning akinesia were underreported by a greater number of physicians than patients. Previous studies report that patients are frequently unaware of dyskinesia, while the caregiver and the physician can notice and observe this presentation [22–24]. Our opposite result may be attributed to cultural differences—involuntary movements are considered more embarrassing by the Japanese population compared with the Western population—and/or the limitation of the short routine examination. Nevertheless, this finding highlights the need for careful and accurate sharing of information between patients and their physicians.

As PD significantly impacts health-related QoL, we used the 8-item Parkinson's Disease Questionnaire (PDQ-8) to assess health-related QoL. As expected, PD had a negative impact on patient QoL, which was, in general, consistently reported by both patients and physicians. Bodily discomfort was poorly recognized by physicians, likely since bodily discomfort is the most subjective of all the questions in PDQ-8. This finding also indicates the importance of physician-patient communication about their symptoms.

The results from our study showed worsened QoL among patients in whom both physician and WOQ-9 assessments were positive but patient self-assessments were negative compared with those in whom physician and WOQ-9 assessments and patient self-assessments all were negative (Supplementary Table 6). These data support the hypothesis that patients often underestimate their WO, which, in turn, worsens their QoL without their awareness.

With respect to morning akinesia, worsened QoL was observed among patients for whom self-awareness was positive but physician assessment was negative, compared with those for whom both self-awareness and physician assessment were negative (Supplementary Table 7). These data indicate that patients who reported morning akinesia had the worst QoL even if physicians did not recognize the symptom. Interestingly, PDQ-8 SI assessed by patients and physicians showed similar scores, which suggests that physicians realized the deterioration in the QoL of patients but did not consider morning akinesia as an attributable reason. As reported previously by our group, morning akinesia significantly affects patient QoL and caregiver burden [25]. Therefore, medical intervention and appropriate gathering of information for morning akinesia are important.

Our study has some limitations. First, there are limitations inherent to these types of surveys, wherein some patients may have been unable to complete the questionnaires because of their physical restrictions. Second, this survey may have been susceptible to responder bias, recall bias, and interviewer bias. Third, while we investigated differences in the perception of WO between patients, their physicians, and based on WOQ-9, the true prevalence of WO in this population is unknown, making data interpretation difficult. Fourth, this survey was conducted in a real-world setting rather than in a research-controlled setting, where questionnaires are usually validated. Thus, there was a lack of control in terms of ensuring that patients understood the questionnaires correctly. In particular, limited patient understanding regarding the symptoms of WO, dyskinesia, and morning akinesia should be acknowledged. Fifth, the lack of information regarding medication use and the severity of motor symptoms assessed by physicians using clinical scales (e.g., Unified Parkinson's Disease Rating Scale (UPDRS), Movement Disorder Society-UPDRS, and Scales for Outcomes in Parkinson's Disease – Motor), as well as the impact of cognitive impairment assessed using clinical scales (Mini-Mental State Examination and Montreal Cognitive Assessment), must also be acknowledged. Sixth, in addition to the well-known limitations of noninterventional and cross-sectional studies, the sample size of our study was relatively small. Seventh, PDQ-8 was used to assess patients' QoL by both patients and physicians, whereas the questionnaire has been designed and validated to be used by patients but not by an external observer without asking the patient to respond to the questionnaire. Lastly, as the focus of the study was on the Japanese population, the generalizability of the results may be limited. Despite these limitations, results from our study emphasize the importance of using effective screening tools, such as WOQ-9, to aid physicians in the diagnosis of WO. Moreover, these findings highlight the need for an open dialog and effective communication and collaboration between patients and physicians. Recent advances in digital technology and biotechnology have led to the development of many types of wearable sensor systems, enabling the continuous long-term monitoring of motor complications [26, 27]. These sensors, which are unobtrusive and accurate, may further assist physicians in diagnosing and managing the symptoms of WO.

## 5. Conclusions

In this study, we investigated the differences in the perception of WO between patients with PD and their physicians in routine clinical practice and found that patients have a low awareness of the symptoms of WO compared with physician assessment and WOQ-9. Conversely, morning akinesia, dyskinesia, and bodily discomfort were underrecognized by physicians. Thus, the use of an objective measure to evaluate WO, such as WOQ-9, combined with improved patient education and awareness of PD treatments and their associated complications is of paramount importance for effective patient-physician communication and to ultimately enhance patient care and treatment outcomes.

## Figures and Tables

**Table 1:** Patient demographics and baseline clinical characteristics from patient questionnaires.

Characteristics	*N* = 235
Age, mean (SD), years	74.0 (9.4)
<65, *n* (%)	31 (13.2)
≥65, *n* (%)	204 (86.8)

Age at PD diagnosis, mean (SD)^*∗*^, years	65.3 (10.8)
Duration of PD, mean (SD), years	8.5 (6.2)
Female, *n* (%)	122 (51.9)

H&Y stage, *n* (%)	
1	27 (11.5)
2	30 (12.8)
3	60 (25.5)
4	63 (26.8)
5	22 (9.4)

Current employment status, *n* (%)	
Full-time	19 (8.1)
Part-time	10 (4.3)
Housekeeping	54 (23.0)
Not working	152 (64.7)
Consultation time, mean (SD), minutes	15.8 (10.8)

H&Y: Hoehn and Yahr; PD: Parkinson's disease; SD: standard deviation. Unknown/missing data are not listed. ^*∗*^When the age was the same as the age at diagnosis, the age at diagnosis was regarded as missing data.

**Table 2:** Physician demographics.

Characteristic	*N* = 92
Age, years, *n* (%)	
≥30 to <40	10 (10.9)
≥40 to <50	29 (31.5)
≥50 to <60	38 (41.3)
≥60	15 (16.3)
Male, *n* (%)	87 (94.6)
No. of patients with PD examined/month, mean (SD)	40.5 (48.3)
Type of hospital or clinic, *n* (%)	
University hospital	6 (6.5)
Public hospital	13 (14.1)
Other hospital	40 (43.5)
Clinic	32 (34.8)
Other	1 (1.1)
Clinical department, *n* (%)	
General internal medicine	17 (18.5)
Neurology	52 (56.5)
Neurosurgery	14 (15.2)
Psychiatry	9 (9.8)
Affiliated academic society, *n* (%)	
Movement Disorder Society of Japan	12 (13.0)
Japanese Society of Neurology	60 (65.2)
The Japanese Association of Rehabilitation Medicine	14 (15.2)
The Japanese Society of Neuropathology	4 (4.3)
None of the above	27 (29.3)

PD: Parkinson's disease; SD: standard deviation.

**Table 3:** Differences in the perception of WO, morning akinesia, and dyskinesia between patients and their physicians.

Total *N* = 235	Patient self-awareness		Physician assessment		WOQ-9 assessed by patients		McNemar's test
	*n*	%	*n*	%	*n*	%	
WO	83	35.3	108	46.0	158	67.2	*p* < 0.0001^*∗*^
Morning akinesia	138	58.7	115	48.9	NA		*p*=0.0032^#^
Dyskinesia	80	34.0	55	23.4	NA		*p*=0.0006^#^

NA: not applicable; WO: wearing-off; WOQ-9: 9-item Wearing-Off Questionnaire. Patients with WOQ-9 scores ≥2 were regarded as having WO. ^*∗*^Physician assessment versus WOQ-9. ^#^Patient self-awareness versus physician assessment.

**Table 4:** Differences in the perception of WO, morning akinesia, and dyskinesia between patient self-awareness and physician assessment.

		Patient self-awareness		McNemar's test
		Yes	No	
WO				*p*=0.0004
Physician assessment	Yes	71 (30.2)	37 (15.7)	
	No	12 (5.1)	115 (48.9)	

Morning akinesia				*p*=0.0032
Physician assessment	Yes	96 (40.9)	19 (8.1)	
	No	42 (17.9)	78 (33.2)	

Dyskinesia				*p*=0.0006
Physician assessment	Yes	41 (17.4)	14 (6.0)	
	No	39 (16.6)	141 (60.0)	

WO: wearing-off. All data are presented as *n* (%).

**Table 5:** Differences in the assessment of WO between patient self-awareness and physician assessment based on patient's WOQ-9.

		Patient WOQ-9		McNemar's test
		Yes	No	
Patient self-awareness	Yes	71 (30.2)	12 (5.1)	*p* < 0.0001
	No	87 (37.0)	65 (27.7)	

Physician assessment	Yes	87 (37.0)	21 (8.9)	*p* < 0.0001
	No	71 (30.2)	56 (23.8)	

WO: wearing-off; WOQ-9: 9-item Wearing-Off Questionnaire. All data are presented as *n* (%). Patients with WOQ-9 scores ≥2 were regarded as having WO.

**Table 6:** Quality of life as assessed by patients and physicians (PDQ-8).

PDQ-8	Patient		Physician		*p* value
	Mean (SD)	Median (min, max)	Mean (SD)	Median (min, max)	
Mobility	60.5 (35.5)	75.0 (0, 100)	62.1 (33.5)	75.0 (0, 100)	0.5812
Activities of daily living	54.4 (36.5)	50.0 (0, 100)	55.4 (35.2)	50.0 (0, 100)	0.4868
Emotional well-being	37.0 (31.4)	25.0 (0, 100)	35.5 (30.2)	25.0 (0, 100)	0.5284
Social support	23.6 (30.8)	0.0 (0, 100)	25.7 (29.6)	25.0 (0, 100)	0.3386
Cognition	33.5 (31.8)	25.0 (0, 100)	31.7 (30.8)	25.0 (0, 100)	0.4577
Communication	33.5 (32.9)	25.0 (0, 100)	30.6 (31.5)	25.0 (0, 100)	0.1115
Bodily discomfort	25.1 (29.8)	25.0 (0, 100)	20.1 (28.4)	0.0 (0, 100)	0.0102
Stigma	28.4 (30.8)	25.0 (0, 100)	30.2 (29.0)	25.0 (0, 100)	0.3281
Summary index	36.9 (24.5)	34.4 (0, 100)	36.4 (24.3)	34.4 (0, 100)	0.7532

PDQ-8: 8-item Parkinson's Disease Questionnaire; SD: standard deviation.

## Data Availability

The data generated and/or analyzed during the current study that were used to support the findings of the study were supplied by AbbVie under license and, therefore, cannot be made freely available. Requests for access to these data should be made to the corresponding author.

## Abbreviations

H&Y: Hoehn and Yahr
PD: Parkinson's disease
PDQ-8: 8-Item Parkinson's Disease Questionnaire
PDQ-8 SI: 8-Item Parkinson's Disease Questionnaire Summary Index
QoL: Quality of life
SD: Standard deviation
UPDRS: Unified Parkinson's Disease Rating Scale
WO: Wearing-off
WOQ-9: 9-Item Wearing-Off Questionnaire
WOQ-19: 19-Item Wearing-Off Questionnaire
WOQ-32: 32-Item Wearing-Off Questionnaire

## Supplementary Materials

Supplementary Materials STROBE checklist. Supplementary Table 1: Study instructions given to the physicians. Supplementary Table 2: Questionnaire items. Supplementary Table 3: Questionnaire for physicians. Supplementary Table 4: Questionnaire for patients. Supplementary Table 5: Duration of motor complications assessed by patients. Supplementary Table 6: Patient demographics and clinical characteristics in subgroups of “wearing-off” based on patient self-awareness and physician assessment and WOQ-9. Supplementary Table 7: Patient demographics and clinical characteristics in subgroups of “morning akinesia” based on patient self-awareness and physician assessment.
