# Supplementary material for: Evaluation of Motor Complications in Parkinson's Disease: Understanding the Perception Gap between Patients and Physicians
Source: Parkinsons Dis. 2021 Dec 22;2021:1599477. doi: 10.1155/2021/1599477 (PMC8716197; doi:10.1155/2021/1599477)
Supplement: Supplementary Materials — STROBE checklist. Supplementary Table 1: Study instructions given to the physicians. Supplementary Table 2: Questionnaire items. Supplementary Table 3: Questionnaire for physicians. Supplementary Table 4: Questionnaire for patients. Supplementary Table 5: Duration of motor complications assessed by patients. Supplementary Table 6: Patient demographics and clinical characteristics in subgroups of “wearing-off” based on patient self-awareness and physician assessment and WOQ-9. Supplementary Table 7: Patient demographics and clinical characteristics in subgroups of “morning akinesia” based on patient self-awareness and physician assessment. [file 1599477.f1.zip › 1599477.f1/Supplementary_Table_1_30JUN21 (1).docx]

Supplementary Table 1: Study instructions given to the physicians.

|  | Physician’s ID: | | |  | |  | Date: | / / | |  |  |
| --- | --- | --- | --- | --- | --- | --- | --- | --- | --- | --- | --- |
|  |  |  |  | |  |  |  |  |  |  |  |
| **1. Please check the following inclusion/exclusion criteria.** | | | | | | | |  |  |  |  |
|  | Inclusion Criteria | | | | | | | | |  |  |
|  | 1. Has the patient been diagnosed with PD? | | | | | | | | |  |  |
|  | Yes | | No | | | →If no, do not include the patient in this study | | | |  |  |
|  | 2. Has the patient received any PD treatment for more than 1 year continuously? | | | | | | | | |  |  |
|  | Yes | | No | | | →If no, do not include the patient in this study | | | |  |  |
|  | 3. Is the patient willing to join this study? | | | | | | | | |  |  |
|  | Yes | | No | | | →If no, do not include the patient in this study | | | |  |  |
|  |  |  |  | |  |  |  |  |  |  |  |
|  | Exclusion Criteria | | | | | | | | |  |  |
|  | 1. Was the patient able to complete the questionnaire by himself/herself? | | | | | | | | |  |  |
|  | Yes | | No | | | →If no, go to next question | | | |  |  |
|  | 2. Has the patient received support to complete questionnaire by his/her caregiver? | | | | | | | | |  |  |
|  | Yes | | No | | | →If no, do not include the patient in this study | | | |  |  |
|  |  |  |  | |  |  |  |  |  |  |  |
| **2. Please obtain informed consent from the patient using the first page of the patient questionnaire.** | | | | | | | | | | | |
|  | Did the patient provide consent and sign the first page of the patient questionnaire? | | | | | | | | |  |  |
|  | Yes | | No | | | →If no, do not include the patient in this study | | | |  |  |
|  |  |  |  | |  |  |  |  |  |  |  |
| **3. Please provide patient questionnaire to patients. Please remind them that they should fill the questionnaire at home and send it back to Anterio Inc. within 14 days.** | | | | | | | | | |  |  |
|  |  |  |  | |  |  |  |  |  |  |  |
| **4. Please fill the date of questionnaire completion.** | | | | | | | |  |  |  |  |
|  | Date: | / / | | |  |  |  |  |  |  |  |
|  |  |  | | |  |  |  |  |  |  |  |
|  | PD: Parkinson’s disease. | | | | | | | | | | |
