# Supplementary material for: Evaluation of Motor Complications in Parkinson's Disease: Understanding the Perception Gap between Patients and Physicians
Source: Parkinsons Dis. 2021 Dec 22;2021:1599477. doi: 10.1155/2021/1599477 (PMC8716197; doi:10.1155/2021/1599477)
Supplement: Supplementary Materials — STROBE checklist. Supplementary Table 1: Study instructions given to the physicians. Supplementary Table 2: Questionnaire items. Supplementary Table 3: Questionnaire for physicians. Supplementary Table 4: Questionnaire for patients. Supplementary Table 5: Duration of motor complications assessed by patients. Supplementary Table 6: Patient demographics and clinical characteristics in subgroups of “wearing-off” based on patient self-awareness and physician assessment and WOQ-9. Supplementary Table 7: Patient demographics and clinical characteristics in subgroups of “morning akinesia” based on patient self-awareness and physician assessment. [file 1599477.f1.zip › 1599477.f1/Supplementary_Table_2_30JUN21 (1).docx]

Supplementary Table 2: Questionnaire items.

| ­­ | Items | Patient | Physician |
| --- | --- | --- | --- |
| Patient general demographics | Age, sex, socioeconomic status, work status | X |  |
| Clinical characteristics | - Age at diagnosis - Disease duration - H&Y staging - Current PD treatment (yes, no) - Patient’s impression in the current presence of (yes/no) - WO - Dyskinesia - Morning akinesia   (If yes, patients to describe its duration)   - Time spent on direct clinical face-to-face interaction with the physicians per visit | X |  |
|  | - Physician’s impression in the current presence of (yes/no) - Loss of medication efficacy - WO - Dyskinesia - Psychiatric complications - Other dopaminergic side effects - Morning akinesia   (If yes, the physician should describe its duration and the clinical parameters that were used in their judgment)   - Time spent on direct clinical face-to-face interaction with the patient per visit |  | X |
| Assessments | WOQ-9 | X |  |
|  | PDQ-8 SI | X | X |
| H&Y: Hoehn and Yahr; PD: Parkinson’s disease; PDQ-8 SI: 8-item Parkinson’s Disease Questionnaire Summary Index; WO: wearing-off; WOQ-9: 9-item Wearing-off Questionnaire. | | | |
