# Supplementary material for: Evaluation of Motor Complications in Parkinson's Disease: Understanding the Perception Gap between Patients and Physicians
Source: Parkinsons Dis. 2021 Dec 22;2021:1599477. doi: 10.1155/2021/1599477 (PMC8716197; doi:10.1155/2021/1599477)
Supplement: Supplementary Materials — STROBE checklist. Supplementary Table 1: Study instructions given to the physicians. Supplementary Table 2: Questionnaire items. Supplementary Table 3: Questionnaire for physicians. Supplementary Table 4: Questionnaire for patients. Supplementary Table 5: Duration of motor complications assessed by patients. Supplementary Table 6: Patient demographics and clinical characteristics in subgroups of “wearing-off” based on patient self-awareness and physician assessment and WOQ-9. Supplementary Table 7: Patient demographics and clinical characteristics in subgroups of “morning akinesia” based on patient self-awareness and physician assessment. [file 1599477.f1.zip › 1599477.f1/Supplementary_Table_3_Revised_12NOV21_clean.docx]

| Supplementary Table 3: Questionnaires for physician. | | | | | | | | | | | | | | | | | | |
| --- | --- | --- | --- | --- | --- | --- | --- | --- | --- | --- | --- | --- | --- | --- | --- | --- | --- | --- |
| Please check before answering the questions => | | | | | | | | | | | | | | Patient No. | | | | |
| <For Doctors> Questionnaire on patients with Parkinson’s disease | | | | | | | | | | | | | | | | | | |
| - This questionnaire survey is owned by “AbbVie GK” and managed by “ANTERIO Inc.” | | | | | | | | | | | | | | | | | | |
| - About the questionnaire | | | | | | | | | | | | | | | | | | |
|  | - As this questionnaire survey maintains anonymity, there is no need to enter your name. | | | | | | | | | | | | | | | | | |
|  | - All answers are processed statistically by computer, and we will never publish personal information in order not to cause inconvenience to the respondents. | | | | | | | | | | | | | | | | | |
|  | - No inquiries shall be made on your personal information provided in the questionnaire. | | | | | | | | | | | | | | | | | |
| - Purpose and use of this questionnaire | | | | | | | | | | | | | | | | | | |
|  | - This questionnaire is intended for doctors who are currently treating Parkinson’s disease. The main purpose is to determine **the treatment situation of patients (Patient Number appears in the upper right).** | | | | | | | | | | | | | | | | | |
|  | - The answers of this questionnaire are used as materials for preparing academic papers to improve the clinical management for future treatment of Parkinson’s disease. | | | | | | | | | | | | | | | | | |
|  | - Analysis results of this questionnaire survey may be used for other purposes such as publication of results by “AbbVie GK” to media after being published as academic papers. | | | | | | | | | | | | | | | | | |
| - Requests when filling in the questionnaire | | | | | | | | | | | | | | | | | | |
|  | - In answering the questions, please answer about the **patient applicable to “Patient No.”** on the upper right. | | | | | | | | | | | | | | | | | |
|  | - Please write clearly with a pen or pencil. | | | | | | | | | | | | | | | | | |
|  | - Please select the answer applicable to each question and circle the number. The number of circles to be marked is stated at the end of the question. | | | | | | | | | | | | | | | | | |
|  | - **For those respondents who selected 1 “Will participate” in Q1 and have completed the questionnaire, please place the questionnaire form in the enclosed reply envelope and post it by XX, April dd, 2018. There is no need to state your name or affix stamps on the envelope.** | | | | | | | | | | | | | | | | | |
|  | - For those who selected 2 “Will not participate,” there is no need to post the questionnaire. | | | | | | | | | | | | | | | | | |
| - If you have any questions about the questionnaire, please contact: | | | | | | | | | | | | | | | | | | |
|  | Questionnaire implementing agency: ANTERIO Inc. (Marketing research agency for INTAGE Inc. group, listed on TSE 1st Section)  ***Monday to Friday from 10 am to 6 pm ・ Toll-free number 0120-xxx-xxx ・ Contact: Maezawa, Tomiuchi** | | | | | | | | | | | | | | | | | |
| Q1 Please read the "Purpose and use of this questionnaire" on the previous page.  Will you participate in this questionnaire survey acknowledging the purpose of this study?  (Select one answer) | | | | | | | | | | | | | | | | | | |
|  | 1 Will participate ⇒ Q2 | | | | | | | | | | 2 Will not participate ⇒ Thank you. | | | | | | | |
| [Questions on Patient’s symptoms] | | | | | | | | | | | | | | | | | | |
| Q2 Would you say that “the effect of medication is not evident” on the Patient as of present?  (Single answer) | | | | | | | | | | | | | | | | | | |
|  | 1 Yes, there is no effect. | | | | | | | | | | | | | | | | | |
|  | 2 Cannot say ⇒ Q4 | | | | | | | | | | | | | | | | | |
|  | 3 No, the effect is evident. ⇒ Q4 | | | | | | | | | | | | | | | | | |
| For Doctors who selected “Yes, there is no effect,” in the above question: | | | | | | | | | | | | | | | | | | |
| Q3 List the reasons why you selected “There is no effect of medication” as specifically as possible. | | | | | | | | | | | | | | | | | | |
|  |  | | | | | | | | | | | | | | | | | |
| Q4 Has “wearing-off” emerged in the Patient as of present? (Single answer) | | | | | | | | | | | | | | | | | | |
|  | 1 Yes | | | | | | | | | | 2 No ⇒ Q6 | | | | | | | |
| For Doctor who selected "Yes" in the above question: | | | | | | | | | | | | | | | | | | |
| Q5 List the reasons why you have selected “wearing-off has emerged" as specifically as possible. | | | | | | | | | | | | | | | | | | |
|  |  | | | | | | | | | | | | | | | | | |
| Q6 Has “dyskinesia” emerged with Patient as of present? (Single answer) | | | | | | | | | | | | | | | | | | |
|  | 1 Yes | | | | | | | | | | 2 No ⇒ Q8 | | | | | | | |
| For Doctors who selected “Yes” in the above question: | | | | | | | | | | | | | | | | | | |
| Q7 List the reasons why you have selected “Dyskinesia has emerged” as specifically as possible. | | | | | | | | | | | | | | | | | | |
|  |  | | | | | | | | | | | | | | | | | |
| Q8 Have “psychological symptoms” emerged in the Patient as of present? (Single answer) | | | | | | | | | | | | | | | | | | |
|  | 1 Yes | | | | | | | | | | 2 No ⇒ Q10 | | | | | | | |
| For Doctors who selected “Yes” in the above question: | | | | | | | | | | | | | | | | | | |
| Q9 List the reasons why you have selected “A psychological symptom has emerged” as specifically as possible. | | | | | | | | | | | | | | | | | | |
|  |  | | | | | | | | | | | | | | | | | |
| Q10 As of present, does the Patient have a "symptom of morning immobility, when the Patient tries to start moving spontaneously in the morning, it takes time, and even if Patient starts to move, s/he can only move slowly?” (Single answer) | | | | | | | | | | | | | | | | | | |
|  | 1 Yes | | | | | | | | | | 2 No ⇒ Q14 | | | | | | | |
| For Doctor who selected “Yes” in the above question: | | | | | | | | | | | | | | | | | | |
| Q11 List the reasons as specifically as possible why you have selected “There is a symptom of morning immobility, when the Patient tries to start to move spontaneously in the morning, it takes time, and even if the Patient starts to move, s/he can only move slowly.” | | | | | | | | | | | | | | | | | | |
|  |  | | | | | | | | | | | | | | | | | |
| Q12 Does the Patient show any adverse reaction to anti-Parkinson’s treatment as of present? (Single answer) | | | | | | | | | | | | | | | | | | |
|  | 1 Yes | | | | | | | | | | 2 No ⇒ Q10 | | | | | | | |
| For Doctors who selected “Yes” in the above question: | | | | | | | | | | | | | | | | | | |
| Q13 List the description of the adverse reaction to anti-Parkinson’s treatment agonist as specifically as possible. | | | | | | | | | | | | | | | | | | |
|  |  | | | | | | | | | | | | | | | | | |
| [For All] | | | | | | | | | | | | | | | | | | |
| Q14 For this Patient, how long is the time required for a single examination on average?  (Enter a numerical value) | | | | | | | | | | | | | | | | | | |
| Approximate time per  examination on average | | | |  | | | | | Min. | | | | | | | | | |
| [PDQ-8 Questionnaire] | | | | | | | | | | | | | | | | | | |
| Q15 Please choose how often the patient experienced following items during the last month according to the information you got from the appointment. If you have no related information, then please choose by your intuition. Please place a single mark in the appropriate number beside each symptom. | | | | | | | | | | | | | | | | | | |
| Have the patients… | | | | | | | Always or cannot do at all | | | Often | | Sometimes | | | | Occasionally | | Never |
| Had difficulty getting around in public? | | | | | | | 1 | | | 2 | | 3 | | | | 4 | | 5 |
| Had difficulty dressing themselves? | | | | | | | 1 | | | 2 | | 3 | | | | 4 | | 5 |
| Felt depressed? | | | | | | | 1 | | | 2 | | 3 | | | | 4 | | 5 |
| Had problems with your close personal relationships? | | | | | | | 1 | | | 2 | | 3 | | | | 4 | | 5 |
| Had problems with your concentration, e.g., when reading or watching TV? | | | | | | | 1 | | | 2 | | 3 | | | | 4 | | 5 |
| Felt unable to communicate with people properly? | | | | | | | 1 | | | 2 | | 3 | | | | 4 | | 5 |
| Had painful muscle cramps or spasms? | | | | | | | 1 | | | 2 | | 3 | | | | 4 | | 5 |
| Felt embarrassed in public due to having Parkinson’s disease? | | | | | | | 1 | | | 2 | | 3 | | | | 4 | | 5 |
| [For All] | | | | | | | | | | | | | | | | | | |
| From here, the questions are intended for the Doctor. | | | | | | | | | | | | | | | | | | |
| * For the following questions, if you have answered for one patient, you will not need to answer for other patients. | | | | | | | | | | | | | | | | | | |
| Q16 How many patients diagnosed with Parkinson’s disease have you treated in the past one month? (Enter a numerical value.) | | | | | | | | | | | | | | | | | | |
| * Please answer on a medical record basis. | | | | | | | | | | | | | | | | | | |
|  | |  | | | patients per month | | | | | | | | | | | | | |
| Q17 Select the main department to which you are affiliated. (Single answer) | | | | | | | | | | | | | | | | | | |
|  | 1 Internal Medicine | | | | | 3 Neurosurgery | | | | | | | | | 5 Others | | | |
|  | 2 Neurology | | | | | 4 Psychiatry | | | | | | | | | ( ) | | | |
| Q18 Select the type of institution where you work. (Single answer) | | | | | | | | | | | | | | | | | | |
|  | 1 Clinic | | | | | 3 National/public hospital | | | | | | | | | 5 Others | | | |
|  | 2 University hospital | | | | | 4 General hospital other than 2&3 | | | | | | | | | ( ) | | | |
| Q19 Select the academic communities to which you are affiliated. (Multiple answers where applicable) | | | | | | | | | | | | | | | | | | |
|  | 1 Movement Disorder Society of Japan | | | | | | | | | | 4 Japanese Society of Neuropathology | | | | | | | |
|  | 2 Japan Society of Neurology | | | | | | | | | | 5 None | | | | | | | |
|  | 3 Japanese Association of Rehabilitation Medicine | | | | | | | | | |  | | | | | | | |
| Q20 Select your sex. (Single answer) | | | | | | | | | | | | | | | | | | |
|  | 1 Male | | | | | | | | | 2 Female | | | | | | | | |
| Q21 Select your age group. (Single answer) | | | | | | | | | | | | | | | | | | |
|  | 1 20s | | 2 30s | | | | | 3 40s | | | | | 4 50s | | | | 5 60+ | |
| This is the end of the questionnaire. Thank you for your cooperation. | | | | | | | | | | | | | | | | | | |
| Lastly, please check again if you have selected “Will participate” in Question 1. | | | | | | | | | | | | | | | | | | |
| After the confirmation, please place this questionnaire in the enclosed reply envelope and post it by XX, April dd, 2018 without affixing stamps. | | | | | | | | | | | | | | | | | | |
