# Supplementary material for: Evaluation of Motor Complications in Parkinson's Disease: Understanding the Perception Gap between Patients and Physicians
Source: Parkinsons Dis. 2021 Dec 22;2021:1599477. doi: 10.1155/2021/1599477 (PMC8716197; doi:10.1155/2021/1599477)
Supplement: Supplementary Materials — STROBE checklist. Supplementary Table 1: Study instructions given to the physicians. Supplementary Table 2: Questionnaire items. Supplementary Table 3: Questionnaire for physicians. Supplementary Table 4: Questionnaire for patients. Supplementary Table 5: Duration of motor complications assessed by patients. Supplementary Table 6: Patient demographics and clinical characteristics in subgroups of “wearing-off” based on patient self-awareness and physician assessment and WOQ-9. Supplementary Table 7: Patient demographics and clinical characteristics in subgroups of “morning akinesia” based on patient self-awareness and physician assessment. [file 1599477.f1.zip › 1599477.f1/Supplementary_Table_4_Revised_12NOV21_clean.docx]

| Supplementary Table 4: Questionnaires for the patient. | | | | | | | | | | | | | | | | | | | | | | | | | | | | | | | | | | | | | | | | | |  |
| --- | --- | --- | --- | --- | --- | --- | --- | --- | --- | --- | --- | --- | --- | --- | --- | --- | --- | --- | --- | --- | --- | --- | --- | --- | --- | --- | --- | --- | --- | --- | --- | --- | --- | --- | --- | --- | --- | --- | --- | --- | --- | --- |
|  | | | | | | | | | | | | | | | | | | | | | | | | | | | | | | | | | | | | | | | | | |  |
|  | | | | | | | | | | | | | | | | | | | Patient No. | | | | | | | | | | | | | | | | | | | | | | |  |
|  | | | | | | | | | | | | | | | | | | |  | | | | | | | | | | | | | | | | | | | | | | |  |
| <For Patients> Questionnaire on the situation of daily life of patients with Parkinson’s disease | | | | | | | | | | | | | | | | | | | | | | | | | | | | | | | | | | | | | | | | | |  |
| - This questionnaire survey is owned by “AbbVie GK” and managed by “ANTERIO Inc.” | | | | | | | | | | | | | | | | | | | | | | | | | | | | | | | | | | | | | | | | | |  |
| - About the questionnaire | | | | | | | | | | | | | | | | | | | | | | | | | | | | | | | | | | | | | | | | | |  |
| - Since this questionnaire survey maintains anonymity, there is no need to enter your name. | | | | | | | | | | | | | | | | | | | | | | | | | | | | | | | | | | | | | | | | | |  |
| - All answers are processed statistically by computer, and we will never publish personal information in order not to cause inconvenience to the respondents. | | | | | | | | | | | | | | | | | | | | | | | | | | | | | | | | | | | | | | | | | |  |
| - No inquiries shall be made on your personal information provided in the questionnaire. | | | | | | | | | | | | | | | | | | | | | | | | | | | | | | | | | | | | | | | | | |  |
| - Purpose and use of this questionnaire | | | | | | | | | | | | | | | | | | | | | | | | | | | | | | | | | | | | | | | | | |  |
| - This questionnaire is intended for patients who are currently receiving treatment for Parkinson’s disease for the purpose of obtaining their opinions on the treatment. | | | | | | | | | | | | | | | | | | | | | | | | | | | | | | | | | | | | | | | | | |  |
| - The answers of this questionnaire are used as materials for preparing academic papers to improve the clinical management for future treatment of Parkinson’s disease. | | | | | | | | | | | | | | | | | | | | | | | | | | | | | | | | | | | | | | | | | |  |
| - Analysis results of this questionnaire may be used for other purposes such as publication of results by “AbbVie GK” to media after being published as academic papers. | | | | | | | | | | | | | | | | | | | | | | | | | | | | | | | | | | | | | | | | | |  |
| - Requests when filling in the questionnaire | | | | | | | | | | | | | | | | | | | | | | | | | | | | | | | | | | | | | | | | | |  |
| - We request that the Patient fill in this questionnaire. If it is difficult for Patient to do so, a family member or other helper may answer on behalf of the Patient. However, please ensure that the Patient’s opinion is entered. | | | | | | | | | | | | | | | | | | | | | | | | | | | | | | | | | | | | | | | | | |  |
| - Please write clearly with a pen or pencil. | | | | | | | | | | | | | | | | | | | | | | | | | | | | | | | | | | | | | | | | | |  |
| - Please select the answer applicable to each question and circle the number. The number of circles to be marked is stated at the end of the question. | | | | | | | | | | | | | | | | | | | | | | | | | | | | | | | | | | | | | | | | | |  |
| - For those respondents who selected 1 “Will participate” in Q1 and have completed the questionnaire, please place the questionnaire in the enclosed reply envelope and post it by XX, April dd, 2018. There is no need to state your name or affix stamps on the envelope. | | | | | | | | | | | | | | | | | | | | | | | | | | | | | | | | | | | | | | | | | |  |
| - For those who selected 2 “Will not participate,” there is no need to post the questionnaire. | | | | | | | | | | | | | | | | | | | | | | | | | | | | | | | | | | | | | | | | | |  |
| - If you have any questions about the questionnaire, please contact: | | | | | | | | | | | | | | | | | | | | | | | | | | | | | | | | | | | | | | | | | |  |
| Questionnaire implementing agency: ANTERIO Inc. (Marketing research agency for INTAGE Inc. group, listed on TSE 1st Section)  *Monday to Friday from 10 am to 6 pm  ・ Toll-free number 0120-xxx-xxx  ・ Contact: Maezawa, Tomiuchi | | | | | | | | | | | | | | | | | | | | | | | | | | | | | | | | | | | | | | | | | |  |
| Q1 Please read the “Purpose and use of this questionnaire” on the previous page. Will you participate in this questionnaire survey acknowledging the purpose of this study? (Single answer) | | | | | | | | | | | | | | | | | | | | | | | | | | | | | | | | | | | | | | | | | |  |
| 1 Will participate ⇒ Q3 | | | | | | | | | | | | 2 Will not participate ⇒ Thank you. | | | | | | | | | | | | | | | | | | | | | | | | | | | | | |  |
| Q2 Select the relationship to the patient. (Single answer) | | | | | | | | | | | | | | | | | | | | | | | | | | | | | | | | | | | | | | | | | |  |
| 1 Patient himself/herself | | | | | | | | | | | | 2 Caregiver | | | | | | | | | | | | | | | | | | | | | | | | | | | | | |  |
| Q3 Please select the number of family members living together, including yourself. (Single answer) | | | | | | | | | | | | | | | | | | | | | | | | | | | | | | | | | | | | | | | | | |  |
| 1 Living alone | 2 2 persons | | | | | | | | | | | 3 3 persons | | | | | | | | | | | | | | | | | | | | | | 4 4+ persons | | | | | | | |  |
| Q4 Select your sex. (Single answer） | | | | | | | | | | | | | | | | | | | | | | | | | | | | | | | | | | | | | | | | | |  |
| 1 Male | | | | | | | | | | | | 2 Female | | | | | | | | | | | | | | | | | | | | | | | | | | | | | |  |
| Q5 State your age. (Enter a numerical value.) | | | | | | | | | | | | | | | | | | | | | | | | | | | | | | | | | | | | | | | | | |  |
| __ years old | | | | | | | | | | | | | | | | | | | | | | | | | | | | | | | | | | | | | | | | | |  |
| Q6 State the age when you were first diagnosed with Parkinson's disease. (Enter a numerical value.) | | | | | | | | | | | | | | | | | | | | | | | | | | | | | | | | | | | | | | | | | |  |
| __ years old | | | | | | | | | | | | | | | | | | | | | | | | | | | | | | | | | | | | | | | | | |  |
| Q7 Select your job before developing Parkinson’s disease. (Single answer) | | | | | | | | | | | | | | | | | | | | | |  | | | **Q8 Select your present job (Single answer)** | | | | | | | | | | | | | | | | | |
| 1 Full-time employee (incl. self-employed) | | | | | | | | | | | | | | | | | | | | | |  | | | 1 Full-time employee (incl. self-employed) | | | | | | | | | | | | | | | | | |
| 2 Part-time employee | | | | | | | | | | | | | | | | | | | | | |  | | | 2 Part-time employee | | | | | | | | | | | | | | | | | |
| 3 Full-time homemaker | | | | | | | | | | | | | | | | | | | | | |  | | | 3 Full-time homemaker | | | | | | | | | | | | | | | | | |
| 4 Other than above and not working | | | | | | | | | | | | | | | | | | | | | |  | | | 4 Other than above and not working | | | | | | | | | | | | | | | | | |
|  | | | | | | | | | | | | | | | | | | | | | | | | | | | | | | | | |  | |  | | | | | | | |
| Q9 Select from the following “Hoehn and Yahr scale” according to the progress stage of your current Parkinson's disease. (Single answer) | | | | | | | | | | | | | | | | | | | | | | | | | | | | | | | | | | | | | | | | | |  |
| 1 Stage 1 | | | | | | | | | | 3 Stage 3 | | | | | | | | | | | | | | | | | | | | | | 5 Stage 5 | | | | | | | | | |  |
| 2 Stage 2 | | | | | | | | | | 4 Stage 4 | | | | | | | | | | | | | | | | | | | | | |  | | | | | | | | | |  |
| [For All] | | | | | | | | | | | | | | | | | | | | | | | | | | | | | | | | | | | | | | | | | |  |
| Q10 Normally, how many hours are you up in a day? (Enter a numerical value.) | | | | | | | | | | | | | | | | | | | | | | | | | | | | | | | | | | | | | | | | | |  |
| Approx. time up in a day: Hr. Min. | | | | | | | | | | | | | | | | | | | | | | | | | | | | | | | | | | | | | | | | | |  |
| Q11 Normally, do you have a symptom such as "arms and legs moving against your intention and interfering with everyday life?” | | | | | | | | | | | | | | | | | | | | | | | | | | | | | | | | | | | | | | | | | |  |
| (Single answer) | | | | | | | | | | | | | | | | | | | | | | | | | | | | | | | | | | | | | | | | | |  |
| 1 Yes | | | | | | | | | | | | 2 No ⇒ Q13 | | | | | | | | | | | | | | | | | | | | | | | | | | | | | |  |
| For Patients who selected "Yes" in the above question: | | | | | | | | | | | | | | | | | | | | | | | | | | | | | | | | | | | | | | | | | |  |
| Q12 How long does the symptom of "arms and legs moving against your intention and interfering with everyday life" persist in a day? Please enter approximate time. (Enter a numerical value.) | | | | | | | | | | | | | | | | | | | | | | | | | | | | | | | | | | | | | | | | | |  |
| Approx. time of the symptom: Hr. Min. | | | | | | | | | | | | | | | | | | | | | | | | | | | | | | | | | | | | | | | | | |  |
| Q13 Normally, do symptoms emerge before the next medicine ("wearing-off phenomenon")? | | | | | | | | | | | | | | | | | | | | | | | | | | | | | | | | | | | | | | | | | |  |
| (Single answer) | | | | | | | | | | | | | | | | | | | | | | | | | | | | | | | | | | | | | | | | | |  |
| 1 Yes | | | | | | | | | | | | 2 No ⇒ Q15 | | | | | | | | | | | | | | | | | | | | | | | | | | | | | |  |
| For Patients who selected "Yes" in the above question: | | | | | | | | | | | | | | | | | | | | | | | | | | | | | | | | | | | | | | | | | |  |
| Q14 State approximate time of the emergence of the symptom before the next medicine (“wearing-off phenomenon”) in a day. (Enter a numerical value.) | | | | | | | | | | | | | | | | | | | | | | | | | | | | | | | | | | | | | | | | | |  |
| Approx. time of the symptom: Hr. Min. | | | | | | | | | | | | | | | | | | | | | | | | | | | | | | | | | | | | | | | | | |  |
| Q15 Normally, do you have a symptom “in the morning, when you try to start to move spontaneously, it takes time, and even if you start to move, you can only move slowly.” | | | | | | | | | | | | | | | | | | | | | | | | | | | | | | | | | | | | | | | | | |  |
| (Single answer) | | | | | | | | | | | | | | | | | | | | | | | | | | | | | | | | | | | | | | | | | |  |
| 1 Yes | | | 2 No ⇒ Q17 | | | | | | | | | | | | | | | | | | | | | | | | | | | | | | | | | | | | | | |  |
| For Patients who selected “Yes” in the above question: | | | | | | | | | | | | | | | | | | | | | | | | | | | | | | | | | | | | | | | | | |  |
| Q16 State the approximate time of the symptom “in the morning, when you try to start to move spontaneously, it takes time, and even if you start to move, you can only move slowly.” | | | | | | | | | | | | | | | | | | | | | | | | | | | | | | | | | | | | | | | | | |  |
| (Enter a numerical value.) | | | | | | | | | | | | | | | | | | | | | | | | | | | | | | | | | | | | | | | | | |  |
| Approx. time of the symptom: Hr. Min. | | | | | | | | | | | | | | | | | | | | | | | | | | | | | | | | | | | | | | | | | |  |
| [For All] | | | | | | | | | | | | | | | | | | | | | | | | | | | | | | | | | | | | | | | | | |  |
| Q17 State the Patient's present level of Support/Care Required. (Single answer） | | | | | | | | | | | | | | | | | | | | | | | | | | | | | | | | | | | | | | | | | |  |
| 1 Not certified for Support/Care | | | | | | | | | | 4 Care Required-1 | | | | | | | | | | | | | | | | | | | | | | 7 Care Required-4 | | | | | | | | | |  |
| 2 Support Required-1 | | | | | | | | | | 5 Care Required-2 | | | | | | | | | | | | | | | | | | | | | | 8 Care Required-5 | | | | | | | | | |  |
| 3 Support Required-2 | | | | | | | | 6 Care Required-3 | | | | | | | | | | | | | | | | | | | | | | | | | | | | | |  | | | |  |
| Q18 a. Select all care services used by the Patient. | | | | | | | | | | | | | | | | | | | | | | | | | | | | | | | | | | | | | | | | | |  |
| * If no service is being used, select 15 “None.” ⇒ Q19 | | | | | | | | | | | | | | | | | | | | | | | | | | | | | | | | | | | | | | | | | |  |
| b. Select the frequency of care service. | | | | | | | | | | | | | | | | | | | | | | | | | | | | | | | | | | | | | | | | | |  |
| c. State duration of time for each service in minutes. | | | | | | | | | | | | | | | | | | | | | | | | | | | | | | | | | | | | | | | | | |  |
| Example: For 2 hours, please state 120 min. | | | | | | | | | | | | | | | | | | | | | | | | | | | | | | | | | | | | | | | | | |  |
|  | | a. | | | | |  | | | | b. Frequency of Service | | | | | | | | | | | | | | | | | | | | | |  | | | c. | | | | |  |  |
|  | | Type of Service | | | | |  | | | | Daily | 5-6 days a week | 3-4 days a week | | | | 1-2 days a week | | | 3 times a month | | | Twice a month | | | Once a month | | Less than once a month | | | | | |  | | | Duration of time for each service (min.) | | | |  |  |
| Day care (rehabilitation) | | 1 | | | | | ⇒ | | | | 1 | 2 | 3 | | | | 4 | | | 5 | | | 6 | | | 7 | | 8 | | | | | | ⇒ | | | M | | | |  |  |
| Day care (follow-up) | | 2 | | | | | ⇒ | | | | 1 | 2 | 3 | | | | 4 | | | 5 | | | 6 | | | 7 | | 8 | | | | | | ⇒ | | | M | | | |  |  |
| Day care (bathing assistance) | | 3 | | | | | ⇒ | | | | 1 | 2 | 3 | | | | 4 | | | 5 | | | 6 | | | 7 | | 8 | | | | | | ⇒ | | | M | | | |  |  |
| Day care (excretion assistance) | | 4 | | | | | ⇒ | | | | 1 | 2 | 3 | | | | 4 | | | 5 | | | 6 | | | 7 | | 8 | | | | | | ⇒ | | | M | | | |  |  |
| Visiting care (rehabilitation) | | 5 | | | | | ⇒ | | | | 1 | 2 | 3 | | | | 4 | | | 5 | | | 6 | | | 7 | | 8 | | | | | | ⇒ | | | M | | | |  |  |
| Visiting care (bathing assistance) | | 6 | | | | | ⇒ | | | | 1 | 2 | 3 | | | | 4 | | | 5 | | | 6 | | | 7 | | 8 | | | | | | ⇒ | | | M | | | |  |  |
| Visiting care (excretion assistance) | | 7 | | | | | ⇒ | | | | 1 | 2 | 3 | | | | 4 | | | 5 | | | 6 | | | 7 | | 8 | | | | | | ⇒ | | | M | | | |  |  |
| Visiting rehabilitation | | 8 | | | | | ⇒ | | | | 1 | 2 | 3 | | | | 4 | | | 5 | | | 6 | | | 7 | | 8 | | | | | | ⇒ | | | M | | | |  |  |
| Transportation service | | 9 | | | | | ⇒ | | | | 1 | 2 | 3 | | | | 4 | | | 5 | | | 6 | | | 7 | | 8 | | | | | | ⇒ | | | M | | | |  |  |
| Assistance tool  (electric bed) | | 10 | | | | |  | | | |  |  |  | | | |  | | |  | | |  | | |  | |  | | | | | |  | | |  | | | |  |  |
| Assistance tool (shower chair) | | 11 | | | | |  | | | |  |  |  | | | |  | | |  | | |  | | |  | |  | | | | | |  | | |  | | | |  |  |
| Assistance tool (wheelchair/walker) | | 12 | | | | |  | | | |  |  |  | | | |  | | |  | | |  | | |  | |  | | | | | |  | | |  | | | |  |  |
| Assistance tool (portable toilet) | | 13 | | | | |  | | | |  |  |  | | | |  | | |  | | |  | | |  | |  | | | | | |  | | |  | | | |  |  |
| Home renovation | | 14 | | | | |  | | | |  |  |  | | | |  | | |  | | |  | | |  | |  | | | | | |  | | |  | | | |  |  |
| None of the above | | 15 | | | | |  | | | |  |  |  | | | |  | | |  | | |  | | |  | |  | | | | | |  | | |  | | | |  |  |
|  | | | | | | | | | | | | | | | | | | | | | | | | | | | | | | | | | | | | | | | | | |  |
| [For All] | | | | | | | | | | | | | | | | | | | | | | | | | | | | | | | | | | | | | | | | | |  |
| [From here on, questions will address the Patient’s treatment for Parkinson’s disease.] | | | | | | | | | | | | | | | | | | | | | | | | | | | | | | | | | | | | | | | | | |  |
| Q19 For a visit to receive the treatment of Parkinson's disease, how long is the time required for a single examination on average? | | | | | | | | | | | | | | | | | | | | | | | | | | | | | | | | | | | | | | | | | |  |
| (Enter a numerical value.) | | | | | | | | | | | | | | | | | | | | | | | | | | | | | | | | | | | | | | | | | |  |
| Approximate time per examination on average Min. | | | | | | | | | | | | | | | | | | | | | | | | | | | | | | | | | | | | | | | | | |  |
| Q20 Do you usually take Levodopa preparation? (Single answer) | | | | | | | | | | | | | | | | | | | | | | | | | | | | | | | | | | | | | | | | | |  |
| 1 Yes | | | 2 No ⇒ Q21 | | | | | | | | | | | | | | | | | | | | | | | | | | | | | | | | | | | | | | |  |
| [WOQ-9 Questionnaire] | | | | | | | | | | | | | | | | | | | | | | | | | | | | | | | | | | | | | | | | | |  |
| In Q13, if you have answered “Yes” for “the symptom emerges before the next medicine” (“wearing-off phenomenon”), please answer the following questions: | | | | | | | | | | | | | | | | | | | | | | | | | | | | | | | | | | | | | | | | | |  |
| If the answer is “No” ⇒ Q22 | | | | | | | | | | | | | | | | | | | | | | | | | | | | | | | | | | | | | | | | | |  |
| Q21 a. Please tick any symptoms that you currently experience during your normal day.  “1 No” or “2 Yes” (Single answer for each item） | | | | | | | | | | | | | | | | | | | | | | | | | | | | | | | | | | | | | | | | | |  |
| b. If you have ticked any of the symptoms, please also tick the box in “b” if that symptom usually improves or disappears after you take a dose of your Parkinson’s medication. (Single answer for each item)  * If you answered “no” in box a, then, there is no need to answer box "b" for the same item. | | | | | | | | | | | | | | | | | | | | | | | | | | | | | | | | | | | | | | | | | |  |
| Symptoms of Parkinson’s disease | | | | a. The following symptoms have occurred at least once a day recently  (Single answer) | | | | | | | | | | | | | | | | | | | | | | |  | | | | | b. After taking levodopa or other anti-Parkinson drugs, such symptoms  (Single answer) | | | | | | | | | |  |
|  | | | | | | | | | | | | | | | | | | | | | | | | | | | | | | | | | | | | | | | | | |  |
| Shaking | | | | | 1 No | | | | | | | | | | | 2 Yes | | | | | | | | | | | | |  | | 1 subside | | | | | | | | 2 Do not change | | |  |
| Slowing down of movement | | | | | 1 No | | | | | | | | | | | 2 Yes | | | | | | | | | | | | |  | | 1 subside | | | | | | | | 2 Do not change | | |  |
| Change in mood or depression | | | | | 1 No | | | | | | | | | | | 2 Yes | | | | | | | | | | | | |  | | 1 subside | | | | | | | | 2 Do not change | | |  |
| Rigidity of muscles | | | | | 1 No | | | | | | | | | | | 2 Yes | | | | | | | | | | | | |  | | 1 subside | | | | | | | | 2 Do not change | | |  |
| Sharp pain or prolonged dull pain | | | | | 1 No | | | | | | | | | | | 2 Yes | | | | | | | | | | | | |  | | 1 subside | | | | | | | | 2 Do not change | | |  |
| Impairment of complex movements of the hand and fingers | | | | | 1 No | | | | | | | | | | | 2 Yes | | | | | | | | | | | | |  | | 1 subside | | | | | | | | 2 Do not change | | |  |
| Difficulty integrating thoughts or slowing down of thought | | | | | 1 No | | | | | | | | | | | 2 Yes | | | | | | | | | | | | |  | | 1 subside | | | | | | | | 2 Do not change | | |  |
| Anxiety or panic attacks | | | | | 1 No | | | | | | | | | | | 2 Yes | | | | | | | | | | | | |  | | 1 subside | | | | | | | | 2 Do not change | | |  |
| Muscle spasms | | | | | 1 No | | | | | | | | | | | 2 Yes | | | | | | | | | | | | |  | | 1 subside | | | | | | | | 2 Do not change | | |  |
|  | | | | | | | | | | | | | | | | | | | | | | | | | | | | | | | | | | | | | | | | | |  |
| [PDQ-8 Questionnaire] | | | | | | | | | | | | | | | | | | | | | | | | | | | | | | | | | | | | | | | | | |  |
| [For All] | | | | | | | | | | | | | | | | | | | | | | | | | | | | | | | | | | | | | | | | | |  |
| Q22 Please choose how often you experienced the following items during the last month.  Please place a single mark in the appropriate number beside each symptom. | | | | | | | | | | | | | | | | | | | | | | | | | | | | | | | | | | | | | | | | | |  |
| Have you… | | | | | | | | | Always or cannot do at all | | | | | Often | | | | | | | | | | Sometimes | | | | | | | | | | | | | Occasionally | | | Never | |  |
| Had difficulty getting around in  public? | | | | | | | | | 1 | | | | | 2 | | | | | | | | | | 3 | | | | | | | | | | | | | 4 | | | 5 | |  |
| Had difficulty dressing yourself? | | | | | | | | | 1 | | | | | 2 | | | | | | | | | | 3 | | | | | | | | | | | | | 4 | | | 5 | |  |
| Felt depressed? | | | | | | | | | 1 | | | | | 2 | | | | | | | | | | 3 | | | | | | | | | | | | | 4 | | | 5 | |  |
| Had problems with your close  personal relationships? | | | | | | | | | 1 | | | | | 2 | | | | | | | | | | 3 | | | | | | | | | | | | | 4 | | | 5 | |  |
| Had problems with your  concentration, e.g., when reading  or watching TV? | | | | | | | | | 1 | | | | | 2 | | | | | | | | | | 3 | | | | | | | | | | | | | 4 | | | 5 | |  |
| Felt unable to communicate with  people properly? | | | | | | | | | 1 | | | | | 2 | | | | | | | | | | 3 | | | | | | | | | | | | | 4 | | | 5 | |  |
| Had painful muscle cramps  or spasms? | | | | | | | | | 1 | | | | | 2 | | | | | | | | | | 3 | | | | | | | | | | | | | 4 | | | 5 | |  |
| Felt embarrassed in public due to  having Parkinson’s disease? | | | | | | | | | 1 | | | | | 2 | | | | | | | | | | 3 | | | | | | | | | | | | | 4 | | | 5 | |  |
|  | | | | | | | | | | | | | | | | | | | | | | | | | | | | | | | | | | | | | | | | | |  |
| Q23 Select your main source of income. (Single answer)  * If multiple answers apply, select the one with the higher income. | | | | | | | | | | | | | | | | | | | | | | | | | | | | | | | | | | | | | | | | | |  |
| 1 Income from work | | 3 Survivor pension | | | | | | | | | | | | | | | | | | | 5 Others | | | | | | | | | | | | | | | | | | | | |  |
| 2 Old-age pension | | 4 Disability pension | | | | | | | | | | | | | | | | | | | 6 No income | | | | | | | | | | | | | | | | | | | | |  |
| Q24 Please state your medical expenses incurred by the treatment of Parkinson’s disease.  * State the sum of the medical expenses to be paid at the medical institution and the cost of medicine. | | | | | | | | | | | | | | | | | | | | | | | | | | | | | | | | | | | | | | | | | |  |
| Approx. amount of ___________ yen per month (Enter a numerical value.) | | | | | | | | | | | | | | | | | | | | | | | | | | | | | | | | | | | | | | | | | |  |
| Q25 Please state your care service expenses.  * State “0” if no care service expense is incurred. | | | | | | | | | | | | | | | | | | | | | | | | | | | | | | | | | | | | | | | | | |  |
| Approx. amount of ___________ yen per month (Enter a numerical value.) | | | | | | | | | | | | | | | | | | | | | | | | | | | | | | | | | | | | | | | | | |  |
| From here, the questions are addressed to those who assist the Patient (Helper). | | | | | | | | | | | | | | | | | | | | | | | | | | | | | | | | | | | | | | | | | |  |
| If there is no Helper, select 1. “No Helper” in Q26 ⇒ Q32 | | | | | | | | | | | | | | | | | | | | | | | | | | | | | | | | | | | | | | | | | |  |
| Q26 Presently, who mainly assists the Patient (Helper)? (Single answer)  *If there are multiple Helpers, select one who spends the longest hours. | | | | | | | | | | | | | | | | | | | | | | | | | | | | | | | | | | | | | | | | | |  |
| 1 No Helper ⇒ Q32 | | | | | | | | | | | | | | | | | | | | | | | | | | | | | | | | | | | | | | | | | |  |
| 2 Spouse | | | | | | 4 Patient’s child | | | | | | | | | | | | | | | | | 6 Relatives, other than 2-5 | | | | | | | | | | | | | | | | | | |  |
| 3 Patient’s parent | | | | | | 5 Sibling | | | | | | | | | | | | | | | | | 7 Helper, caregiver, nurse | | | | | | | | | | | | | | | | | | |  |
|  | | | | | |  | | | | | | | | | | | | | | | | | 8 Others (　　　　　　　　　　　　　　) | | | | | | | | | | | | | | | | | | |  |
| Q27 Select the sex of the person who mainly assists the Patient (Helper). (Single answer) | | | | | | | | | | | | | | | | | | | | | | | | | | | | | | | | | | | | | | | | | |  |
| 1 Male | | | | | | | | | | | | | | | | | | | | | | | | | | | | | 2 Female | | | | | | | | | | | | |  |
| Q28 Does the person who mainly assists the Patient (Helper) live with Patient? (Single answer) | | | | | | | | | | | | | | | | | | | | | | | | | | | | | | | | | | | | | | | | | |  |
| 1 Yes | | | | | | | | | | | | | | | | | | | | | | | | | | | | | 2 No | | | | | | | | | | | | |  |
| Q29 Select the job of the person who mainly assists the Patient (Helper) before the Patient developed Parkinson’s disease.  (Single answer) | | | | | | | | | | | | | | |  | | | **Q30 Select the present job of the person who mainly assists the Patient (Helper).**  **(Single answer)** | | | | | | | | | | | | | | | | | | | | | | | |  |
| 1 Full-time employee | | | | | | | | | | | | | | |  | | | 1 Full-time employee | | | | | | | | | | | | | | | | | | | | | | | |  |
| 2 Part-time employee | | | | | | | | | | | | | | |  | | | 2 Part-time employee | | | | | | | | | | | | | | | | | | | | | | | |  |
| 3 Full-time homemaker | | | | | | | | | | | | | | |  | | | 3 Full-time homemaker | | | | | | | | | | | | | | | | | | | | | | | |  |
| 4 Other than above and not working | | | | | | | | | | | | | | |  | | | 4 Other than above and not working | | | | | | | | | | | | | | | | | | | | | | | |  |
|  | | | | | | | | | | | | | | | | | | | | | | | | | | | | | | | | | | | | | | | | | |  |
| Q31 Select the main source of income of the person who mainly assists the Patient (Helper). (Single answer)  * If multiple answers apply, select the one with the higher income. | | | | | | | | | | | | | | | | | | | | | | | | | | | | | | | | | | | | | | | | | |  |
| 1 Income from work | | | | | | | | | 3 Survivor pension | | | | | | | | | | | | | | | | | | | | | 5 Others | | | | | | | | | | | |  |
| 2 Old-age pension | | | | | | | | | 4 Disability pension | | | | | | | | | | | | | | | | | | | | | 6 No income | | | | | | | | | | | |  |
| [For All] | | | | | | | | | | | | | | | | | | | | | | | | | | | | | | | | | | | | | | | | | |  |
| Q32 State the relationship between the Patient and the person who filled in this questionnaire. (Single answer) | | | | | | | | | | | | | | | | | | | | | | | | | | | | | | | | | | | | | | | | | |  |
| 1 Patient | | | | | | | | | | 4 Patient’s child | | | | | | | | | | | | | | | | | | | | | | 7 Helper, caregiver, nurse | | | | | | | | | |  |
| 2 Spouse | | | | | | | | | | 5 Sibling | | | | | | | | | | | | | | | | | | | | | | 8 Others | | | | | | | | | |  |
| 3 Patient’s parent | | | | | | | | | | 6 Relative other than 2-5 | | | | | | | | | | | | | | | | | | | | | |  | | | | | | | | | |  |
| This is the end of the questionnaire. Thank you for your cooperation.  Lastly, please check again if you have selected "Will participate" in Question 1. | | | | | | | | | | | | | | | | | | | | | | | | | | | | | | | | | | | | | | | | | |  |
| After your confirmation, please place this questionnaire in the enclosed reply envelope and post it by XX, April dd, 2018 without affixing stamps. | | | | | | | | | | | | | | | | | | | | | | | | | | | | | | | | | | | | | | | | | |  |
