# Supplementary material for: Evaluation of Motor Complications in Parkinson's Disease: Understanding the Perception Gap between Patients and Physicians
Source: Parkinsons Dis. 2021 Dec 22;2021:1599477. doi: 10.1155/2021/1599477 (PMC8716197; doi:10.1155/2021/1599477)
Supplement: Supplementary Materials — STROBE checklist. Supplementary Table 1: Study instructions given to the physicians. Supplementary Table 2: Questionnaire items. Supplementary Table 3: Questionnaire for physicians. Supplementary Table 4: Questionnaire for patients. Supplementary Table 5: Duration of motor complications assessed by patients. Supplementary Table 6: Patient demographics and clinical characteristics in subgroups of “wearing-off” based on patient self-awareness and physician assessment and WOQ-9. Supplementary Table 7: Patient demographics and clinical characteristics in subgroups of “morning akinesia” based on patient self-awareness and physician assessment. [file 1599477.f1.zip › 1599477.f1/Supplementary_Table_5_Revised_12NOV21 (1).docx]

Supplementary Table 5: Duration of motor complications assessed by patients.

| Total N=235 | | Duration of motor complication (hours/day) | |
| --- | --- | --- | --- |
|  | | n | Mean (SD) |
| WO | | 80 | 2.95 (2.52) |
| Morning akinesia | | 135 | 1.32 (1.82) |
| Dyskinesia | | 78 | 3.47 (4.00) |
| SD: standard deviation; WO: wearing-off. |  |  |  |
