# Supplementary material for: Evaluation of Motor Complications in Parkinson's Disease: Understanding the Perception Gap between Patients and Physicians
Source: Parkinsons Dis. 2021 Dec 22;2021:1599477. doi: 10.1155/2021/1599477 (PMC8716197; doi:10.1155/2021/1599477)
Supplement: Supplementary Materials — STROBE checklist. Supplementary Table 1: Study instructions given to the physicians. Supplementary Table 2: Questionnaire items. Supplementary Table 3: Questionnaire for physicians. Supplementary Table 4: Questionnaire for patients. Supplementary Table 5: Duration of motor complications assessed by patients. Supplementary Table 6: Patient demographics and clinical characteristics in subgroups of “wearing-off” based on patient self-awareness and physician assessment and WOQ-9. Supplementary Table 7: Patient demographics and clinical characteristics in subgroups of “morning akinesia” based on patient self-awareness and physician assessment. [file 1599477.f1.zip › 1599477.f1/Supplementary_Table_6_Revised_12NOV21_clean.docx]

Supplementary Table 6: Patient demographics and clinical characteristics in subgroups of “wearing-off” based on patient self-awareness and physician assessment and WOQ-9.

| Demographic | Patient self-awareness/physician assessment of “wearing-off” | | | | | | | |
| --- | --- | --- | --- | --- | --- | --- | --- | --- |
|  | +/+ | | +/− | | −/+ | | −/− | |
|  | WOQ-9 | | | | | | | |
|  | + | − | + | − | + | − | + | − |
| n (%) | 60 (25.5) | 11 (4.7) | 11 (4.7) | 1 (0.4) | 27 (11.5) | 10 (4.3) | 60 (25.5) | 55 (23.4) |
| Age, mean (SD), years | 71.0 (9.9) | 75.5 (6.2) | 73.8 (7.7) | 78.0 (NA) | 73.9 (9.2) | 73.9 (9.2) | 74.6 (7.9) | 76.2 (11.0) |
| <65, n (%) | 12 (20.0) | 0 (0.0) | 0 (0.0) | 0 (NA) | 4 (14.8) | 1 (10.0) | 6 (10.0) | 8 (14.5) |
| ≥65, n (%) | 48 (80.0) | 11 (100.0) | 11 (100.0) | 1 (NA) | 23 (85.2) | 9 (90.0) | 54 (90.0) | 47 (85.5) |
| Age at PD diagnosis,  mean (SD)*, years | 59.3 (12.1) | 63.8 (11.1) | 65.9 (11.8) | 74.0 (NA) | 65.8 (9.1) | 65.2 (9.6) | 68.5 (8.3) | 68.2 (10.6) |
| Duration of PD,  mean (SD), years | 11.2 (6.2) | 11.7 (7.6) | 7.9 (7.9) | 4.0 (NA) | 8.6 (4.3) | 9.6 (7.3) | 6.1 (4.3) | 7.6 (6.7) |
| Sex, female, n (%) | 38 (63.3) | 7 (63.6) | 8 (72.7) | 1 (100) | 17 (63.0) | 3 (30.0) | 19 (31.7) | 29 (52.7) |
| H&Y stage, n (%) |  |  |  |  |  |  |  |  |
| 1 | 1 (1.7) | 0 (0.0) | 1 (9.1) | 1 (100) | 2 (7.4) | 1 (10.0) | 15 (25.0) | 6 (10.9) |
| 2 | 1 (1.7) | 0 (0.0) | 1 (9.1) | 0 (0.0) | 5 (18.5) | 2 (20.0) | 12 (20.0) | 9 (16.4) |
| 3 | 19 (31.7) | 1 (9.1) | 3 (27.3) | 0 (0.0) | 5 (18.5) | 3 (30.0) | 16 (26.7) | 13 (23.6) |
| 4 | 30 (50.0) | 6 (54.5) | 4 (36.4) | 0 (0.0) | 5 (18.5) | 1 (10.0) | 9 (15.0) | 8 (14.5) |
| 5 | 5 (8.3) | 1 (9.1) | 1 (9.1) | 0 (0.0) | 2 (7.4) | 1 (10.0) | 5 (8.3) | 7 (12.7) |
| Current employment status, n (%) |  |  |  |  |  |  |  |  |
| Full-time | 7 (11.7) | 0 (0.0) | 0 (0.0) | 0 (0.0) | 1 (3.7) | 1 (10.0) | 5 (8.3) | 5 (9.1) |
| Part-time | 1 (1.7) | 0 (0.0) | 1 (9.1) | 0 (0.0) | 1 (3.700) | 1 (10.0) | 5 (8.3) | 1 (1.8) |
| Housekeeping | 18 (30.0) | 4 (36.4) | 6 (54.5) | 0 (0.0) | 5 (18.5) | 0 (0.0) | 9 (15.0) | 12 (21.8) |
| Not working | 34 (56.7) | 7 (63.6) | 4 (36.4) | 1 (100) | 20 (74.1) | 8 (80.0) | 41 (68.3) | 37 (67.3) |
| Consultation time,  mean (SD), minutes | 14.9 (8.6) | 16.8 (16.0) | 20.9 (12.0) | 10.0 (NA) | 15.4 (9.4) | 14.5 (6.9) | 16.0 (12.1) | 16.0 (11.6) |
| PDQ-8 SI  (assessed by patient) | 52.0 (23.4) | 44.8 (14.1) | 41.5 (25.2) | 18.8 (NA) | 37.7 (19.1) | 34.2 (17.5) | 30.5 (24.2) | 25.6 (23.0) |
| PDQ-8 SI  (assessed by physician) | 50.1 (22.7) | 50.0 (17.3) | 33.2 (25.7) | 15.6 (NA) | 39.7 (21.1) | 39.1 (19.4) | 26.5 (22.3) | 28.5 (23.8) |
| +, present; -, absent; H&Y: Hoehn and Yahr; NA: not applicable; PD: Parkinson’s disease; SD: standard deviation; PDQ-8 SI: 8-item Parkinson’s Disease Questionnaire Summary Index; WOQ-9: 9-item Wearing-off Questionnaire.  Unknown/missing data are not listed.  *When the age was the same as the age at diagnosis, the age at diagnosis was regarded as missing data. | | | | | | | | |
