# Supplementary material for: Evaluation of Motor Complications in Parkinson's Disease: Understanding the Perception Gap between Patients and Physicians
Source: Parkinsons Dis. 2021 Dec 22;2021:1599477. doi: 10.1155/2021/1599477 (PMC8716197; doi:10.1155/2021/1599477)
Supplement: Supplementary Materials — STROBE checklist. Supplementary Table 1: Study instructions given to the physicians. Supplementary Table 2: Questionnaire items. Supplementary Table 3: Questionnaire for physicians. Supplementary Table 4: Questionnaire for patients. Supplementary Table 5: Duration of motor complications assessed by patients. Supplementary Table 6: Patient demographics and clinical characteristics in subgroups of “wearing-off” based on patient self-awareness and physician assessment and WOQ-9. Supplementary Table 7: Patient demographics and clinical characteristics in subgroups of “morning akinesia” based on patient self-awareness and physician assessment. [file 1599477.f1.zip › 1599477.f1/Supplementary_Table_7_Revised_12NOV21_clean.docx]

Supplementary Table 7: Patient demographics and clinical characteristics in subgroups of “morning akinesia” based on patient self-awareness and physician assessment.

| Demographic | Patient self-awareness/physician assessment of “morning akinesia” | | | |
| --- | --- | --- | --- | --- |
|  | +/+ | +/− | −/+ | −/− |
| n (%) | 96 (40.9) | 42 (17.9) | 19 (8.1) | 78 (33.2) |
| Age, mean (SD), years | 74.4 (8.9) | 73.2 (8.1) | 72.7 (9.0) | 74.2 (10.7) |
| <65, n (%) | 10 (10.4) | 3 (7.1) | 3 (15.8) | 15 (19.2) |
| ≥65, n (%) | 86 (89.6) | 39 (92.9) | 16 (84.2) | 63 (80.8) |
| Age at PD diagnosis, mean (SD)*, years | 64.6 (11.3) | 64.3 (11.0) | 63.5 (9.1) | 67.3 (10.5) |
| Duration of PD, mean (SD), years | 9.6 (6.2) | 9.0 (7.0) | 9.8 (6.7) | 6.6 (5.1) |
| Sex, female, n (%) | 56 (58.3) | 24 (57.1) | 11 (57.9) | 31 (39.7) |
| H&Y stage, n (%) |  |  |  |  |
| 1 | 3 (3.1) | 1 (2.4) | 0 (0.0) | 23 (29.5) |
| 2 | 5 (5.2) | 4 (9.5) | 3 (15.8) | 18 (23.1) |
| 3 | 25 (26.0) | 16 (38.1) | 3 (15.8) | 16 (20.5) |
| 4 | 41 (42.7) | 10 (23.8) | 4 (21.1) | 8 (10.3) |
| 5 | 12 (12.5) | 3 (7.1) | 3 (15.8) | 4 (5.1) |
| Current employment status, n (%) |  |  |  |  |
| Full-time | 4 (4.2) | 2 (4.8) | 2 (10.5) | 11 (14.1) |
| Part-time | 3 (3.1) | 1 (2.4) | 1 (5.3) | 5 (6.4) |
| Housekeeping | 25 (26.0) | 12 (28.6) | 4 (21.1) | 13 (16.7) |
| Not working | 64 (66.7) | 27 (64.3) | 12 (63.2) | 49 (62.8) |
| Consultation time, mean (SD), minutes | 16.4 (11.6) | 16.9 (9.0) | 15.1 (15.7) | 14.7 (9.3) |
| PDQ-8 SI (assessed by patient) | 47.9 (22.1) | 39.9 (23.1) | 30.7 (20.8) | 23.5 (22.0) |
| PDQ-8 SI (assessed by physician) | 45.1 (22.1) | 43.2 (25.8) | 41.8 (19.9) | 20.7 (19.3) |

+, present; -, absent; H&Y, Hoehn and Yahr; PD, Parkinson’s disease; PDQ-8 SI: 8-item Parkinson’s Disease Questionnaire Summary Index; SD, standard deviation.

Unknown/missing data are not listed.

*When the age was the same as the age at diagnosis, the age at diagnosis was regarded as missing data.
